# Allele mining and enhanced genetic recombination for rice breeding

**DOI:** 10.1186/s12284-015-0069-y

**Published:** 2015-11-25

**Authors:** Hei Leung, Chitra Raghavan, Bo Zhou, Ricardo Oliva, Il Ryong Choi, Vanica Lacorte, Mona Liza Jubay, Casiana Vera Cruz, Glenn Gregorio, Rakesh Kumar Singh, Victor Jun Ulat, Frances Nikki Borja, Ramil Mauleon, Nickolai N. Alexandrov, Kenneth L. McNally, Ruaraidh Sackville Hamilton

**Affiliations:** Plant Breeding Genetics and Biotechnology Division and International Rice Research Institute, Los Banos, Philippines; T.T. Chang Genetic Resource Center, International Rice Research Institute, Los Banos, Philippines

**Keywords:** Disease resistance, Genetic diversity, MAGIC, SNP database

## Abstract

Traditional rice varieties harbour a large store of genetic diversity with potential to accelerate rice improvement. For a long time, this diversity maintained in the International Rice Genebank has not been fully used because of a lack of genome information. The publication of the first reference genome of Nipponbare by the International Rice Genome Sequencing Project (IRGSP) marked the beginning of a systematic exploration and use of rice diversity for genetic research and breeding. Since then, the Nipponbare genome has served as the reference for the assembly of many additional genomes. The recently completed 3000 Rice Genomes Project together with the public database (SNP-Seek) provides a new genomic and data resource that enables the identification of useful accessions for breeding. Using disease resistance traits as case studies, we demonstrated the power of allele mining in the 3,000 genomes for extracting accessions from the GeneBank for targeted phenotyping. Although potentially useful landraces can now be identified, their use in breeding is often hindered by unfavourable linkages. Efficient breeding designs are much needed to transfer the useful diversity to breeding. Multi-parent Advanced Generation InterCross (MAGIC) is a breeding design to produce highly recombined populations. The MAGIC approach can be used to generate pre-breeding populations with increased genotypic diversity and reduced linkage drag. Allele mining combined with a multi-parent breeding design can help convert useful diversity into breeding-ready genetic resources.

## Introduction

Genetic diversity is the foundation for crop improvement. Traditional varieties or landraces of rice harbour a large store of valuable genes that can be used to develop new varieties with improved yield potential, higher nutritional quality, and higher tolerance of the stresses of future climate. The recent completion of re-sequencing of 3,000 genebank accessions (The 3000 Rice Genomes Project, 2014) has revealed the allelic diversity of multiple rice genomes. The sequence data provides a “digital library” of the 3,000 accessions, thus presenting opportunities to identify natural genetic variations in rice. The published SNP database (Alexandrov et al. 2014) has enabled the community to identify rice germplasm carrying favorable alleles simply by data mining. However, the use of landraces for breeding is often hampered by linkage drag. To make use of the diversity, an efficient mating design is needed to break the undesirable linkages and to convert landraces to breeding-ready genetic resources.

Multi-parent Advanced Generation InterCross (MAGIC) is a breeding method to produce highly recombined germplasm (Cavanagh et al. 2008; Bandillo et al. 2013; Mackay et al. 2014; Huang et al. 2015). The increased recombination in MAGIC populations can lead to novel rearrangements of alleles and greater genotypic diversity. Inclusion of elite varieties together with the landraces in such crosses can produce pre-breeding materials with reduced linkage drag. This breeding method can be used to generate a gene pool enriched in essential traits.

In this paper, we will highlight the contributions of the benchmark reference genomes to the exploration of rice diversity for breeding. We will use disease resistance traits to illustrate the extraction of useful germplasm from the 3,000 sequenced accessions and propose a breeding scheme to accumulate multiple favorable traits in a breeding-ready genetic background.

## Review

### Use of genetic diversity and the Rice Genebank

Since the 1960s, rice improvement has gone through distinct stages defined by the incorporation of different sets of traits. First, the introduction of the dwarfing gene (*sd1*) changed the plant architecture, producing the first semi-dwarf variety, IR8. The short stature of IR8 allowed fertilizer input without succumbing to lodging. Thus, modifying plant type resulted in a dramatic increase in yield that marked the beginning of the Green Revolution. This was followed by improvement in disease and insect resistance in the 1970s. Bacterial blight resistance gene *Xa4* was used in nearly all IRRI-bred varieties, resulting in successful control of bacterial blight for many years. In the mid- 1980s, IR64 was released as a variety with broad adaptability, multiple disease resistance and good eating quality. IR64 is considered the most popular variety ever grown, covering more than 9 million hectares of irrigated riceland at one time. In the 1990s increased attention was given to the less favourable, rainfed environment that limited production because of abiotic stresses, such as too much or too little water, and multiple soil problems. Since then, new genes conferring tolerance to submergence, drought, salinity, and phosphorus (P)-deficient soils have been identified in landraces and transferred to modern varieties. For example, submergence tolerance in FR13A (IRGC#117267), and salinity tolerance in Pokkali (IRGC#108921) have been actively used in breeding programs (Singh and Flowers, 2010,Thomson et al. 2010; Mackill et al. 2012).

**Fig. 1:**
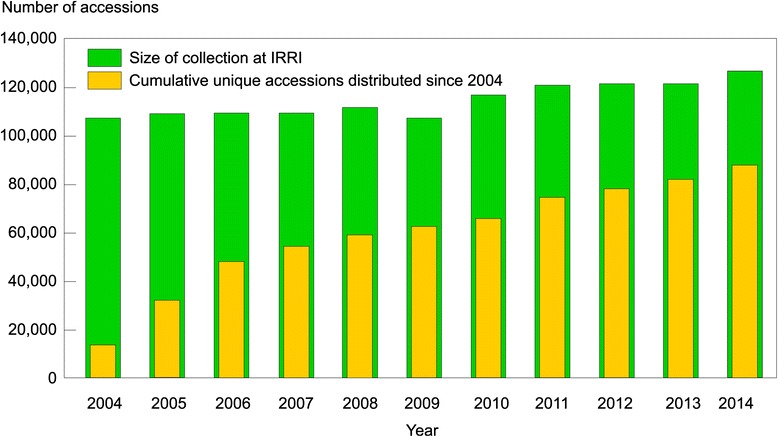
Growing needs for new diversity by breeders and global scientists

Nearly all of these breeding achievements started with extracting diversity from the landraces. A large store of this diversity is maintained in the International Rice Genebank (http://irri.org/our-work/seeds) that holds the world’s largest collection of domesticated rice *Oryza sativa* and *O. glaberrima* accessions. Currently, the Genebank has about 124,000 accessions of *O. sativa* and 6,000 accessions of wild rice species. It is considered the world’s best managed Genebank which provides a well curated and conserved resource for the global research community. The Genebank is widely used as shown by the growing request for accessions over the past 10 years (Fig. 1).

Yet, the wealth of diversity maintained in the Genebank is largely untapped. Based on analysis of the pedigrees of modern varieties, it is estimated that only about 5 % of the genebank accessions have been used in the development of modern varieties. Several reasons account for this. Landraces often have undesirable traits that require a long process of crossing to break the linkage to those traits. The passport data of Genebank accessions have information on their geographic origins and some have basic morphological and agronomic information collected when they are planted in the field. But, besides these, there is limited information to guide the selection of appropriate materials as donors of new traits for breeding. Identifying good donors for breeding is therefore difficult solely based on phenotypic data or passport data. This method also has serious limitations because the effects of individual genes could be masked by others.

The success of breeding has also resulted in the reduction of genetic diversity in modern varieties. The wide adaptability of mega-varieties has influenced breeding programs towards using a narrow pool of germplasm with good agronomic performance records. For example, many breeding programs tend to concentrate on incorporating large-effect genes into mega-varieties, such as IR64 in Southeast Asia, Swarna, Sambha Mahsuri, MTU1010 in India, and BR11 in Bangladesh. Although this breeding strategy has been successful in alleviating key production constraints, the unintended consequence is that many current varieties in Southeast Asia and South Asia have a relatively narrow genetic base. The question is whether upgrading mega - varieties with major genes is sufficient to address future constraints caused by climate change and to meet consumer demands for different markets.

### Significance of the first *O. sativa* reference genome

Recognizing the need to explore the genetic diversity of rice, the International Rice Genome Sequencing Project (IRGSP) was initiated in 1997 (Eckardt 2000). This global effort eventually produced the best assembled genome of *O. sativa* japonica cultivar Nipponbare (International Rice Genome Sequencing Project 2005). It is fitting to reflect on how the first gold standard genome has led to genome investigation and expanding applications. Feuillet et al (2011) showed that, since the publication of the Nipponbare genome in 2005, there has been a dramatic increase in QTL cloning in rice relative to other cereals. With a high-quality reference genome available, rice becomes a model for genomic data tagged with annotations for exploring sequences in other crop species. Development in rice sequence analysis also serves as a prototype for other crops.

However, given the deep diversity of *O. sativa*, a single genome is not sufficient to reveal the genomic diversity of rice. Additional *de novo* genome assembly and high-density genotyping efforts were started to uncover the diversity. Several *O. sativa* accessions were sequenced at sufficient depth for the construction of new draft genomes: *93-11* an indica genome with 12 chromosomes and ~ 39 megabases (mb) of unmapped scaffolds (Yu et al. 2005; Gao et al. 2013); Kasalath, an aus genome with 12 chromosomes and ~43 mb of unmapped scaffolds (Sakai et al. 2014); IR64, an indica genome with 2,919 scaffolds; and DJ123, an aus genome with 2,819 scaffolds (Schatz et al. 2014). These new reference genomes allow the discovery of candidate alleles controlling important agronomic traits that are not present in the Nipponbare genome (e.g. the *Sub1* locus in FR13A). Many more published and unpublished studies together have genotyped over 2000 accessions to-date using array-based or re-sequencing platforms (Table 1 lists published studies). In all cases, the Nipponbare genome has served as the benchmark for high-quality reference genome. Although these new genomes have served genetic research well, they are not adequate for rice breeding that has to take a population genomics approach using genotypes from a large number of rice accessions.

**Table 1:** Studies investigating multiple rice genomes using different sequencing and genotyping platforms

Sequencing and genotyping platform	Number of lines	Nature of germplasm	Number of high-quality SNPs discovered	Reference
Map-based sequencing	1	Single japonica variety	NA	International Rice Genome Sequencing Project (2005)
Perlegen chip array	20	OryzaSNP set: diverse collection mostly from japonica and indica, some from aus, deepwater, and aromatic group, actively used in international breeding programs	~160 k	McNally et al. 2009
Affymetrix single nucleotide polymorphism (SNP) array	413	Diverse rice varieties from 82 countries	44,100	Zhao et al. 2011
Illumina GAx resequencing	1,083 cultivated + 446 wild rice	Cultivated indica and japonica, wild rice *O. rufipogon*	~8 million	Huang et al. 2012
Illumina GA2 resequencing	50	Mostly indica and japonica, *O. rufipogon*, *O. nivara*	~6.5 millon	Xu et al. 2012
Illumina GoldenGate BeadArray 768-plex and 384-plex	180	Japan improved and landrace accessions (temperate japonica)	2,688	Yonemaru et al. 2012
Illumina HiSeq2000	529	Parental lines from IRRI breeding program; USDA rice genebank minicore subset	~6.5 million	Agrama et al. 2009, Yu et al. 2003, Zhao et al. 2014
Illumina GA2 resequencing	3,000	Primarily landraces and released varieties	~30 million	The 3,000 Rice Genomes Project 2014
Illumina HiSeq2000	54	21 elite cultivars from CIAT rice breeding program; 33 elite cultivars from U.S. rice breeding program	~18 million	Duitama et al. 2015

### The 3K rice genomes project

Through a collaboration among the Chinese Academy of Agricultural Sciences, the Beijing Genomics Institute-Shenzhen (BGI-Shenzhen), and IRRI, 3,000 germplasm accessions have been sequenced at an average depth of 14X (The 3000 Rice Genomes Project, 2014). The sequence data of the 3000 genomes were aligned with the reference genome Nipponbare. This resulted in the identification of approximately 20 million SNPs. The large amount of data is organized into a SNP-Seek database (Alexandrov et al. 2014; www.oryzasnp.org/iric-portal). The SNP-Seek database provides a user-friendly resource to explore the genetic diversity of a large collection of germplasm accessions. Through this database, one can query by SNP haplotypes, germplasm accession names, passport data, and basic agronomic data. Thus, SNP-Seek database offers a simple and easy-access platform to search for new alleles from the 3K accessions. The SNP data will evolve over time through alignment of the sequences to additional high-quality reference genomes.

To facilitate global collaboration and effective use of the new genomic resources, IRRI initiated the International Rice Informatics Consortium (IRIC) in 2013 (http://iric.irri.org). The objectives of the IRIC are to: 1) organize available genotyping, phenotyping, expression and other available data for rice germplasm into a linked, consistent, and reliable source of information for the global research community; 2) provide user-friendly access to browse, search, and analyze the data through a single portal; and 3) support information sharing, public awareness and capacity building.

Through this collaborative consortium, the 3K rice genomes data can serve as a community-wide resource enabling the exploration of rice diversity by the global community. The challenge ahead is associating phenotypes with the sequenced accessions. Below, we will use disease resistance traits to illustrate the identification of new diversity from the traditional varieties or landraces.

### Allele mining

**Fig. 2:**
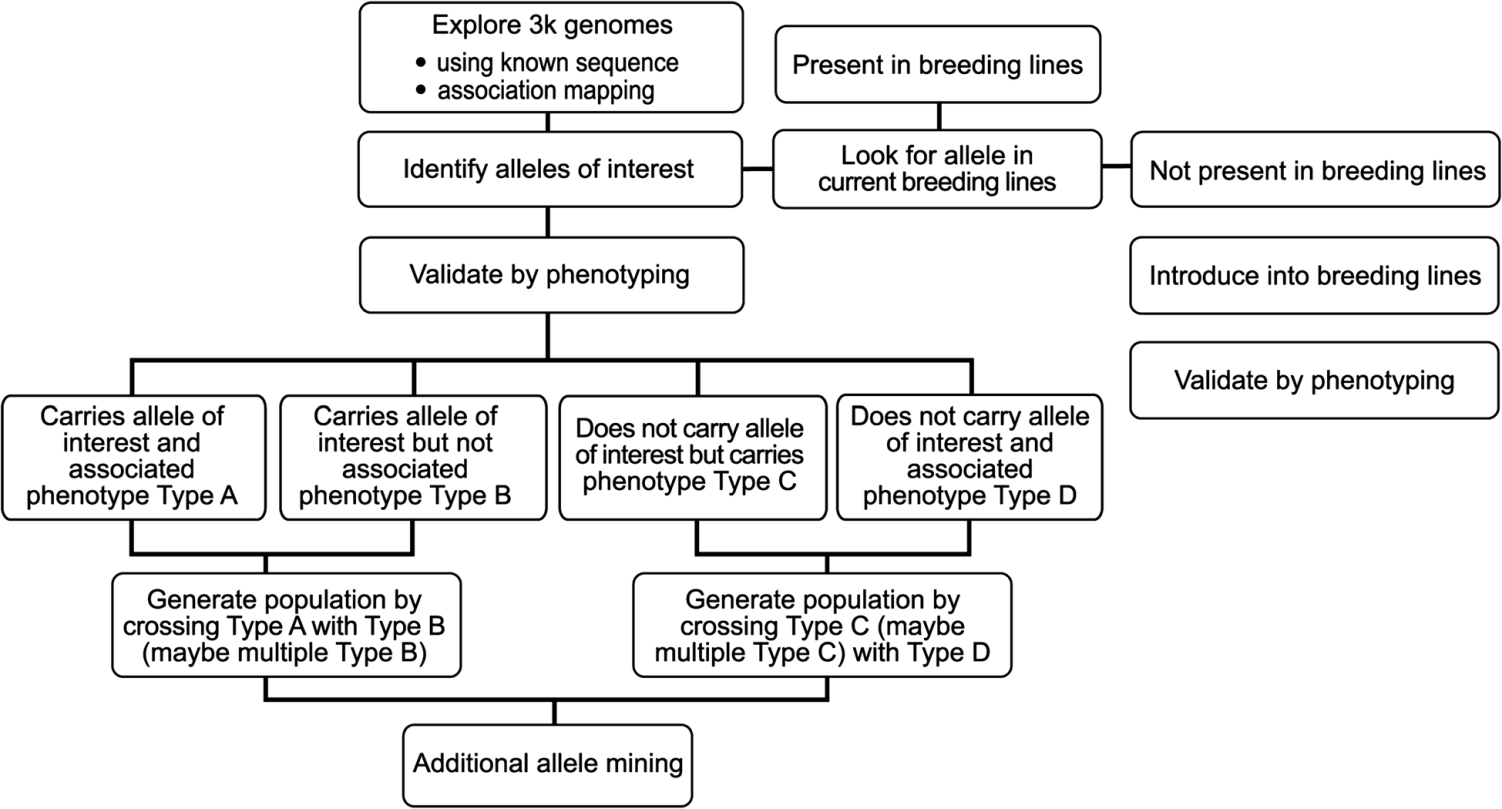
Allele mining work flow. Explore the sequenced genomes using known sequences or haplotypes. Identify accessions and evaluate phenotypes. Test the presence of new alleles in breeding lines and initiate crossing as needed. Mapping of traits and testing in diverse genetic background. Phenotype validation and new crosses to identify additional alleles

The main objective of allele mining is to identify a small number of genomes and accessions from a large collection based on either sequence or phenotypic information. The genome data provides a digital filter to identify a small set of accessions for targeted phenotyping. The 3K genomes dataset provides an efficient means to map traits and validate certain target sequences. Known sequences can be used to search the genome database, and identify accessions with unique sequences and haplotypes. Figure 2 summarizes the main steps of allele mining to explore and use the dataset.

### Increased diversity for disease resistance

In rice, a large number of resistance genes have been identified for major diseases such as blast and bacterial blight. However, because of the evolution of pathogen populations and the dynamic nature of host-pathogen interaction, a constant search for diverse resistance mechanisms is needed to manage disease epidemics as well as new emerging diseases. With the available 3K genomes data, it is possible to identify new diversity for disease resistance through data mining. Using multiple disease resistance as target traits, we have identified accessions with potentially new resistance genes in the 3K genomes.

### Case 1: Mining diversity of blast resistance genes

Rice blast caused by *Magnaporthe oryzae* is a perpetual problem of rice production. Although major successes have been achieved for controlling rice blast, this effort has been hampered by the rapid adaptation of the pathogens. As of now, more than 25 blast resistance genes (*Pi*) have been documented using different cloning methods. These genes are distributed within 16 loci, suggesting that *Pi* genes from different donors tend to be located within the same genomic locus; for example, *Pi2*/*Pi9*/*Piz-t*, *Pi5*/*Pii*, and *Pik*/*Pi1*/*Pikm*/*Pikh*/*Pik*. Except for *pi21*, *Pid2* and *Pi54*, most *Pi* genes exclusively encode proteins or their variants containing nucleotide binding site (NBS) and leucine-rich repeat (LRR) domains, suggesting that the NBS-LRR gene family constitutes the main reservoir of *Pi* genes in rice. Most of these NBS-LRR gene loci consist of multiple gene members, representing a complex genomic structure commonly conserved in other plant species. It is worth noting that the *Pi* alleles in the same locus from different donor plants are located either in the same genomic position (orthologues) or in different positions (paralogues). For example, *Piz-t* and *Pi2* are located in the same positions approximately 22-kb apart from *Pi9* in the respective clusters (Qu et al. 2006; Zhou et al. 2006). A similar genomic organization was documented for the different *R* genes in the *Pish* locus. *Pish* and *Pi35* are considered as orthologues since they are located in the same genomic positions (Takahashi et al. 2010; Fukuoka et al. 2014). In contrast, they are considered as paralogues to *Pi37* and *Pi64* since each of them is located in different positions in the respective clusters (Lin et al. 2007; Takahashi et al. 2010; Fukuoka et al. 2014; Ma et al. 2015).

By comparing the differences in sequence and structure in resistant and susceptible haplotypes, blast *R*-gene loci can be grouped into two types. A type I locus refers to the one in which high sequence similarity and conserved genomic organization are maintained between resistant and susceptible haplotypes. For this type, the differentiation between *R* and *S* alleles is primarily caused by localized mutations, including nucleotide substitutions, small insertions/deletions (InDels), and insertion of transposable elements. In contrast, a type II locus refers to one in which genomic organization or sequence similarity or both in resistant haplotypes are significantly different from that in susceptible haplotypes. It is therefore more feasible to develop *R*-gene specific markers to distinguish functional from non-functional alleles for a type II locus than for a type I locus. An understanding of the evolutionary differentiation of resistance genes is therefore important for exploring additional diversity in the rice gene pool.

Analyses of cultivar-specific sequences that are not in the Nipponbare reference genome revealed the presence of NBS-LRR-coding sequences in some of the 3,000 genomes, suggesting that type II *R-*gene loci are frequently distributed in rice genomes. Functional characterization of these novel type II *R-*gene loci could help to identify more functional *R* genes. Using three type II *R-*gene loci, *Pi9*, *Pi5*, and *Pikm*, as targets, approximately 23.6 %, 31.3 %, and 33.4 % of the 3,000 genomes were found to carry the putative functional *Pi9*, *Pi5*, and *Pikm* alleles, respectively. Furthermore, 75 % of the lines bearing the predicted *Pi*9-allele belong to the *indica* type, a frequency almost four times higher than in *japonica* type. The association of *R* genes within rice types is of interest for the exploration and deployment of *R* gene diversity in different geographic regions. The shortlisted lines containing putative functional *R*-gene alleles should also expedite the identification of novel alleles with broad-spectrum resistance to diverse blast isolates.

### Case 2. *In silico* prediction, allele mining, and transcriptional profiling to identify genes involved in bacterial blight resistance

Bacterial blight (BB), caused by *Xanthomonas oryzae* pv. *oryzae* (*Xoo*), is the most important bacterial disease of rice. At least 39 resistance genes (*Xa*) have been identified from wild and cultivated accessions. Resistance to *Xoo* has a strong race-specific component but in contrast to other systems (e.g., the blast pathogen), a very small number of the R proteins contain NBS-LRR domains. Quite often those genes have been classified into distinct functional categories that include transcription factors, membrane transporters, or genes involved in miRNA stability (Boch et al. 2014).

These distinct resistance mechanisms probably have evolved in response to the unusual interaction with the pathogen. *Xoo* delivers a unique type of protein that modulates transcription status of the host cell. The bacteria use the so called Transcription Activator-like Effectors (TALEs) to recognize specific Effector Binding Elements (*EBEs*) in the target promoter of the host gene. TALE-mediated activation of the target genes supports pathogen proliferation, hence making them as susceptibility genes (*S*). Therefore, it is not surprising that selection pressure to escape *Xoo* colonization has targeted the EBE site itself as well as the transcription machinery. For instance, resistance gene *xa13* is caused by a mutation disrupting the effector binding affinity of the native *S* gene *Os8N3*. Other resistance genes, such as *Xa10* or *Xa23*, appear to have decoy *EBEs* upstream of executor genes, which explains the localized cell death phenotype in the plant. On the other hand, the recessive resistance gene *xa5* is an allele of the general transcription factors TFIIAγ (Iyer and McCouch 2004), which probably confer resistance by modulating activation of other TALE targets.

Two independent research groups broke the DNA recognition code of TALEs (Boch et al. 2009; Moscou and Bogdanove 2009), thus opening the door for *in silico* target identification within the rice genome (Noël et al. 2013). By using pathogen TALE information, it is now possible to generate a catalogue of potential target genes using the 3,000 rice genomes. Allele mining exercises together with rapid phenotyping can be used to identify effective *R-*gene combinations for breeding purposes or to assess quantitative contribution of natural variants in overall resistance. As an example, a search within a reported EBE site of major *S*-gene promoters, such as members of the SWEET sucrose-efflux transporter family, yielded a set of candidate accessions (Table 2). The frequency of mutations across accessions (0.2 % to 11 %) varies widely among target genes. Most of the mutations are distributed across all rice groups with no clear pattern of association. However, one exception is a mutation that is found in low frequency (0.08 %) and involves two genotypes from the aus population. A larger sample of aus accessions and functional validation are needed to support this observation.

**Table 2:** Single nucleotide polymorphism (SNP) found among 2494 rice lines at EBE sites targeted by known TAL effectors

Gene name	Locus name	Chr	SNP ID	Reference/alternate allele	Frequency of alternate allele in diversity panel (%)
*SWEET 11* (*Os8N3*)	LOC_Os08g42350	8	26728868	A/G	0.2
*SWEET 14* (*Os11N3*)	LOC_Os11g31190	11	18174486	G/A	11.2
	LOC_Os11g31190	11	18174499	G/C	0.08
	LOC_Os11g31190	11	18174555	C/A	0.2
*SWEET 13* (*Os12N3*)	LOC_Os12g29220	12	17305906	T/G	7.5
	LOC_Os12g29220	12	17305913	G/C	7.1

To validate gene function, the expression level of candidate genes in the selected accessions can be used to associate gene activation and phenotype. In some cases artificially designed TALEs have been used to dissect the interaction and to activate specific target genes, thus avoiding the problem of collateral targets. The 3K genomes can serve as training materials to generate accurate TALE-based algorithm prediction and more precise TALE engineering. The community is now positioned to discover resistance and susceptibility genes involved in *Xoo*-rice interaction by using a combination of TALE target prediction, allele mining, and transcriptome profiling.

### Case 3: Mining virus resistance alleles

At least 16 virus species have been reported to infect rice (Hibino 1996; Zhou et al. 2013). A majority of rice viruses are distributed in Asia, but some viruses such as *Rice yellow mottle virus* (RYMV), *Rice hoja blanca virus*, and *Rice giallume virus* also cause serious damages to rice production on other continents (Hibino 1990). Genes involved in resistance to three rice viruses, *Rice stripe virus* (RSV) (Wang et al. 2014), *Rice tungro spherical virus* (RTSV) (Lee et al. 2010), and RYMV (Albar et al. 2006; Orjuela et al. 2013), have been identified and used in developing rice varieties withstanding damages caused by the viruses.

Rice tungro virus disease caused by the interaction between RTSV and *Rice tungro bacilliform virus* (RTBV) is one of the most prevalent virus diseases of rice in South and Southeast Asia (Hibino 1996). Several dozen rice germplasm accessions were found to show resistance to RTSV, but those showing resistance to RTBV appeared to be very limited (Hibino 1990). Resistance to RTSV is a recessive trait controlled by a gene (*tsv1*, LOC_Os07g36940) encoding translation initiation factor 4 gamma (eIF4G) (Lee et al. 2010). The SNP and deletion that are associated with RTSV resistance were found to be located upstream of the sequences encoding a signature middle domain of eIF4G (Lee et al. 2010). Examination of the 3,000 rice genomes for the SNP and deletion associated with RTSV resistance indicated that about 5 % of the sequenced accessions have the resistance alleles of *tsv1* (Table 3). The frequency of the rice accessions presumably resistant to RTSV may increase if other SNPs in the same region are found to be also associated with RTSV resistance.

**Table 3:** Numbers of *Oryza sativa* accessions with SNPs and deletions associated with virus resistance among the 3,000 sequenced accessions

Gene name	Locus name^a^	SNP/deletion associated with phenotype^b^						Expected phenotype^c^	Number of corresponding accessions among 3,000 accessions (%)	Reference
	LOC_Os07g36940	3167								
*tsv1*		T	A	T	G	T	T	S to RTSV	2,821 (94)	Lee et al. (2010)
		T	**G**	T	G	T	T	R to RTSV	78 (2.6)	
		T	A	T	G	**C**	T	R to RTSV	21 (0.7)	
		T	A	T	**−**	**−**	**−**	R to RTSV	52 (1.7)	
		Others^d^						Uncertain	28 (0.9)	
		727								
*STV11*	LOC_Os11g30910	G	C	G	G	C	G	S to RSV	2,798 (93.3)	Wang et al. (2014)
		**−**	**−**	**−**	**−**	**−**	**−**	R to RSV	10 (0.3)	
		Others^d^						Uncertain	192 (6.4)	
		925								
*RYMV1*	LOC_Os04g42140	G	A	A	A	T	A	S to RYMV	3,000 (100)	Albar et al. (2006)
		**A**	A	A	A	T	A	R to RYMV	0(0)	

^a^From Michigan State University’s Rice Genome Annotation Project Release 7 (Os-Nipponbare-Reference-IRGSP-1.0 (Kawahara et al. 2013)

^b^Numbers above the first nucleotides indicate the positions in the coding sequences of the corresponding genes. Nucleotides (underlined bold) and deletions (−) are those reported to be associated with resistance to the corresponding viruses

^c^S: susceptible, R: resistant, RTSV: Rice tungro spherical virus, RSV: Rice stripe virus, RYMV: Rice yellow mottle virus

^d^Sequences with other SNPs or deletions, and uncertain sequences

RSV causes chronic yield losses in japonica rice cultivated in East Asian countries such as China, Korea, and Japan (Hibino 1996). The QTL linked to resistance to RSV have been identified in several indica rice cultivars such as Modan and Kasalath (Wang et al. 2014). The RSV resistance in Kasalath is a dominant trait, and it was found to be controlled by a gene (*STV11*, LOC_Os11g30910) encoding a sulphotransferase that catalyzes the conversion of salicylic acid to sulphonated salicyclic acid. Examination of about 300 accessions of *O. sativa* and *O. rufipogon* revealed that RSV resistance is associated with a 6-nucleotide deletion in the middle of *STV11*, and that the resistance allele of *STV11* is predominantly found in indica rice and *O. rufipogon* accessions from South and Southeast Asia (Wang et al. 2014). Examination of the 3,000 rice accessions for the 6-nucleotide deletion in *STV11* revealed that ten accessions (0.3 %) carry the deletion, and are potentially resistant to RSV (Table 3).

RYMV causes serious damage in African rice (*O. glaberrima*), and appears to be widely distributed on the African continent (Hibino 1996; Albar et al. 2006). Resistance alleles of two genes, *RYMV1* (LOC_Os04g42140) encoding an isoform of translation initiation factor 4 gamma (eIF(iso)4G) and *RYMV2* (corresponding to LOC_Os01g68970 in *O. sativa*) encoding a homolog of constitutive expression of PR genes 5, were found to confer resistance to RYMV (Albar et al. 2006; Orjuela et al. 2013). All resistance alleles of *RYMV1* and *RYMV2* were found in several accessions of *O. glaberrima*, except one resistance allele of *RYMV1* from indica rice cultivar Gigante. The sequence comparison of the *RYMV1* alleles between RYMV-resistant Gigante and susceptible variety IR64 revealed that a single SNP is associated with the resistance to RYMV (Albar et al. 2006). Examination of the 3,000 rice accessions for the SNP associated with RYMV resistance in Gigante showed that none of the 3,000 *O. sativa* accessions carry the SNP associated with RYMV resistance, suggesting that RYMV resistance is an extremely rare trait in *O. sativa*.

Mining for alleles associated with virus resistance among the 3,000 rice accessions showed that about 5.3 % of the accessions are potentially resistant to at least one of the rice viruses, suggesting that considerable diversity is available for breeding of virus-resistant rice varieties. The number of the accessions potentially resistant to rice viruses may increase with further identification of genes and SNP associated with resistance to RSV, RTSV, RYMV, and other rice viruses in the future.

### Case 4. Brown spot

Brown spot caused by *Bipolaris oryzae* is a growing problem in South Asia and Southeast Asia. The disease is endemic, causing a chronic yield loss. Disease expression is complex as incidence and severity are affected by drought, soil and nutrient conditions. A previous study using a bi-parental cross [landrace Denorado (resistant) x IR36 (susceptible)] reported a QTL on chromosome 12 (Banu et al 2008). By screening with a single virulent isolate of *B. oryzae,* this QTL was confirmed in a multi-parent population (indica MAGIC) and a 2,000 diversity panel using the association mapping approach. The QTL with significant SNP markers spanning a 1.3 Mb region on chromosome 12 was identified. Within this region, local haplotypes, consisting of 7 SNPs were associated with resistance. The 3K panel was explored to validate this QTL in a diverse background. The SNP haplotype was used to identify accessions carrying the resistant haplotype in the 3K genomes. The identified haplotype was present in 2777 (93 %) of the 3000 accessions. It seems surprising that the R haplotype is so prevalent considering the absence of active selection for brown spot resistance in most breeding programs. A possible explanation is that landraces or traditional varieties were selected for disease resistance or other traits tightly linked to this region by farmers long ago before modern rice breeding; thus most landraces carry the R-haplotype. This idea seems to be supported by examining the region using the QTL genome viewer (Yonemaru et al. 2010) which shows presence of QTLs for 1000 grain weight, blast disease and soil stress tolerance. A similar example is the conservation of *Pup1*, a gene for enhanced phosphorous uptake, in upland and drought tolerant varieties (Chin et al. 2011). The *Pup1* gene is observed in high frequency in the absence of targeted selection. Furthermore, brown spot resistance may not necessarily be conferred by a single locus. Phenotyping will be needed to validate whether the identified accessions are resistant to brown spot. Accessions not predicted by these hypotheses can be further examined for other genomic regions that control the trait as shown in Fig. 2.

The above case studies illustrate that the frequency of detecting predictive haplotypes varies with traits. This can be due to a number of reasons. It may reflect the history of selection in the population of landraces and their geographic origins. Some alleles can be naturally rare, occurring only at low frequencies, and a large population of sequenced genomes is needed to detect them. Such a contrasting case is shown by virus resistance. In contrast to the relatively high frequency of resistance haplotype to RTSV (5 %), resistance haplotype to RYMV is not detected in the 3K genomes. It could be a case of extremely low frequency or because RYMV resistance can be found only in *O. glaberrima*. So far, the examples presented are all major genes with large effects. The challenge is to apply allele mining for complex traits controlled by multiple QTLs, each with a small effect.

### Converting landrace diversity to breeding resources

**Fig. 3:**
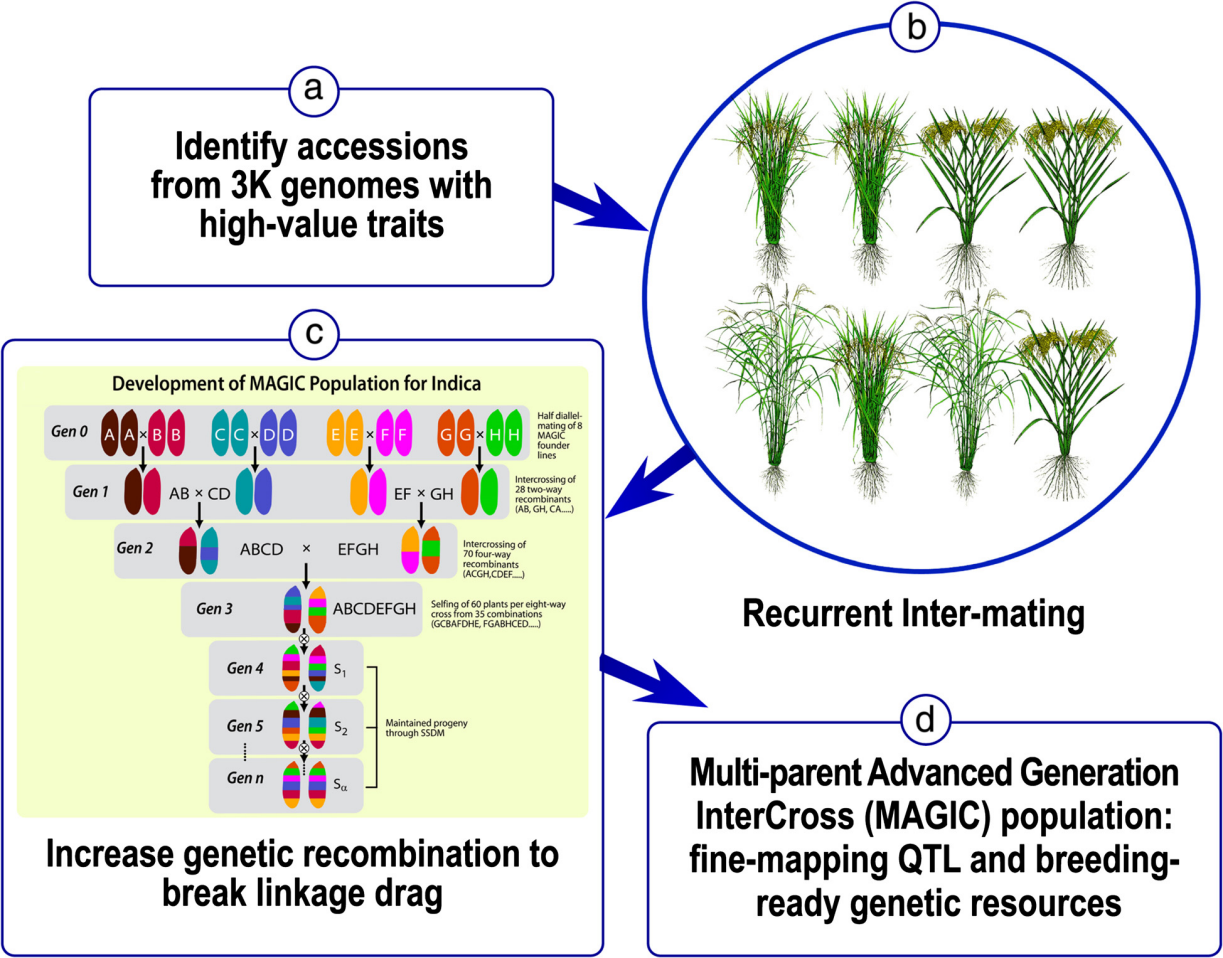
Mating design to convert genetic diversity to genetic resources with concentration of high-value traits. The design involves **a**) identifying new accessions with useful traits validated by phenotyping, **b**) using new accessions together with an elite line to produce a MAGIC population, **c**) breaking unfavorable linkages (adopted from Bandillo et al. 2013.), and **d**) producing breeding-ready resources

The allele mining exercises suggest that it is possible to identify new accessions for targeted phenotyping. Once validated phenotypically, the accessions can be used as donors to incorporate new diversity into breeding lines (Fig. 3). The conventional approach is to backcross the selected accessions to elite breeding lines and create bi-parental segregating populations. Plants carrying the target traits are further backcrossed to elite genetic backgrounds. Backcrossing has been the standard approach for introducing diversity into elite germplasm; however, given the large number of accessions expected from allele mining, alternative breeding designs should be considered in the conversion of landrace diversity into breeding resources. Below, we propose a breeding scheme to harness multiple favourable loci.

Multiparent Advanced Generation InterCross (MAGIC) is a breeding method that involves several cycles of inter-mating among multiple parental lines (Cavanagh et al. 2008). In a standard MAGIC design, founder lines (from 4 to 16) are inter-crossed in a half-diallele design. The F_1_ plants are then inter-crossed to produce new F_1_s with four different genomes (four-way cross). The resulting F_1_ is crossed with other F_1_s in eight-way crosses. The F_1_ plants from 8-ways are then selfed by single seed decent for several generations to produce Advanced Inbred Lines (AIL). The populations resulting from such a breeding design are expected to have increased recombination and generate pre - breeding materials with new genotypic diversity (Bandillo et al 2013). Breeders have adopted variations of this design in an attempt to maximise recombinations.

The MAGIC design offers an alternative to conventional bi-parental and advanced backcross methods. It is particularly suitable for converting landrace germplasm into elite breeding materials. The multiple cycles of meiosis is expected to break the tight linkages between favourable and unfavourable loci, hence reducing linkage drag. Further, through multiple recombinations, increased genotypic diversity will lead to enhanced transgressive segregation. Through cycles of inter-mating, multiple traits are recombined resulting in an accumulation of favourable genes in the same process.

Figure 3 illustrates the scheme of converting landraces to breeding–ready genetic resources. Selected germplasm accessions identified from allele mining are used as founders. At least one popular variety is added to the founder pool to provide an elite genetic background. After completing the mating cycles of two-way, four-way, and eight-way crosses, the plants are selfed to fixation. The fixed lines serve as a permanent mapping population for fine mapping and extraction of favourable gene combinations. One can envision the creation of such modular gene pools for high- priority traits with new combinations of favourable alleles. Based on this concept, two new MAGIC populations have been produced, one for heat tolerance (Changrong Ye, IRRI, personal communication) and one for disease and insect resistance.

Given the relatively small size of MAGIC populations (1,000-1,500 lines), they are amenable for sequencing and multi-location testing (Verbyla et al. 2014). By exposing these populations to diverse conditions and environments, novel gene-phenotypes relationships can be discovered and extracted for the regular breeding programs. Every test location has the potential of revealing new genes and traits unique to the environment. Further, the phenotype-genotype associations, pleiotropic and epistatic interactions revealed in MAGIC populations can be used as a training set in developing predictive models for genomic selection (Scutari et al. 2014). Integrated analysis of the genetic populations and breeding lines will lead to the discovery of agronomically important genes and favorable GxE interactions.

While MAGIC populations offer a number of advantages for the extraction of new alleles, additional breeding approaches should be considered depending on breeding objectives. For introducing simple traits, marker assisted backcrossing (MABC) has been widely used to improve a single genotype by adding a favourable donor allele. In the case of complex traits, however, MABC becomes complicated as multiple donor alleles have to be introgressed to obtain the desired phenotype. Marker-assisted recurrent selection (MARS) is an improvement over MABC. Essentially, MARS involves marker-aided selection (MAS) of lines followed by inter-mating to derive recombined lines carrying multiple donor alleles of interest. Phenotypic recurrent selection with no MAS has been used as an alternate approach, although this could be a time-consuming process. For both MAGIC and MARS, careful selection of parental lines is important to capture multiple favourable traits. Unlike multi-allelic MARS, the selection of MAGIC parents is not guided by known QTLs but by their phenotypes. This increases the potential of capturing novel QTLs with small phenotypic variance and QTL combinations to enhance the improvement of complex traits. An insightful review of different backcrossing and breeding approaches is provided by Ribaut and Ragot (2007).

## Conclusions

The development of climate-resilient rice is ever more important in the coming decades as extreme climatic conditions will become more frequent across the globe. Effects of climate change will certainly present new environmental stresses that impact food production.

Allelic and genotypic diversity are both essential to meet the demand for rice varieties that are tolerant of adverse climatic conditions and at the same time have high productivity and nutritional value. It is imperative to have an efficient system to explore genetic diversity and deliver it to breeding.

Since the release of the reference genome of Nipponbare, new advances in genome sequencing technologies have made it feasible to reveal the genome diversity of many genetic populations. The sequenced 3,000 genomes have demonstrated the potential of exploring the diversity of the Genebank at IRRI. The revealed diversity in the 3,000 genomes represents only a small portion of the potential diversity available in over 124,000 accessions stored in the Genebank. Further sequencing of the landrace germplasm alongside the existing breeding collection is needed to have a comprehensive view of the diversity available for rice improvement.

After allele mining, an essential step is to channel the diversity to genetic populations that can be used readily by breeding. The MAGIC approach can be adopted to build pre-breeding populations accessible to breeding programs. Although more time and efforts may be required to develop multiple MAGIC populations, the potential benefits are large. Relative to bi-parental populations, MAGIC populations will have greater genotypic diversity, a higher level of recombination, and reduced linkage drag. Because of these advantages, the MAGIC approach has been applied to many crop and plant species for genetic research and breeding (Huang et al. 2015). Rice, with its deep diversity, can benefit from combining allele mining and novel mating designs to develop new genetic resources for breeding.
